# Case Report: A Pseudoaneurysm Involving the Left Common Iliac Artery Secondary to Brucellosis: A Rare Case Report

**DOI:** 10.3389/fbioe.2021.811229

**Published:** 2021-12-13

**Authors:** Qilong Wang, Liang Tang, Yue Qin, Qi Wang, Ping Zhang, Zhihua Cheng

**Affiliations:** ^1^ Department of Vascular Surgery, The First Bethune Hospital of Jilin University, Changchun, China; ^2^ Department of Neurology, Songyuan Jilin Oilfield Hospital, Songyuan, China; ^3^ Department of Hepatopancreatobiliary Surgery, The First Bethune Hospital of Jilin University, Changchun, China

**Keywords:** brucellosis, common iliac artery, pseudoaneurysm, endovascular surgery, case report

## Abstract

Pseudoaneurysms of the common iliac artery caused by Brucellosis are exceedingly uncommon. Infected common iliac artery pseudoaneurysms, particularly those caused by brucellosis, are more difficult to diagnose and cure than general pseudoaneurysms. The risk of mortality is significantly high in this condition. Nonsurgical treatment of a brucellosis-induced common iliac artery pseudoaneurysm is futile, and it should be operated on as soon as feasible. Long-term and multi-course antibacterial therapy with combination antibiotics is required. For the treatment of *Brucella*-infected pseudoaneurysms, endovascular surgery can be both effective and safe.

## Introduction

Brucellosis is a zoonotic infection caused by bacteria of the genus *Brucella* (Wang et al., 2017; Amjadi et al., 2019; Bagheri Nejad et al., 2020; Iqbal et al., 2020). Fever, hyperhidrosis, fatigue, joint soreness, and lymphadenopathy (Jia et al., 2017; Liang et al., 2018; Alkahtani et al., 2020) are some of the clinical manifestations of the condition. Infection is spread mostly through infected animals and their products (Zhang et al., 2018; Willems et al., 2021). *Brucella* infection in humans is typically transmitted by direct contact with the skin and mucous membranes (Haque et al., 2011). However, on occasion, food-borne diseases are transferred through the digestive tract and infections are transmitted through the respiratory tract via inhalation of contaminated droplets and dust; humans are generally susceptible to *Brucella*. Additionally, brucellosis can have a detrimental effect on the respiratory, circulatory, digestive, and nervous systems. Endocarditis and pericarditis are the most prevalent disease symptoms of the circulatory system, while pseudoaneurysms involving the left common iliac artery are extremely rare. A pseudoaneurysm involving the common iliac artery that is infected is unstable. In medicine, it is frequently referred to as an “untimed bomb” implanted in the body that is likely to “explode” at any time, referring to aneurysm rupture and hemorrhage. Severe bleeding can result in hemorrhagic shock and lead to death. Herein, we report a rare instance of pseudoaneurysm affecting the left common iliac artery due to brucellosis and detail the treatment of this patient.

## Case Presentation

**FIGURE 1:**
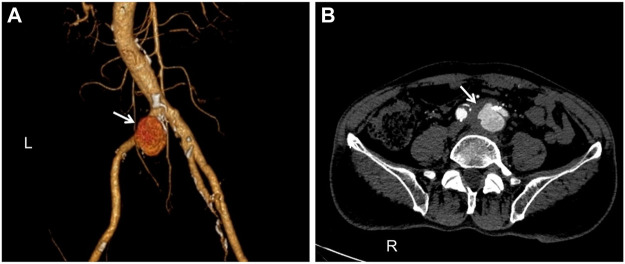
Computed tomography angiography (CTA): **(A)**. The pseudoaneurysm has a maximum diameter of 33 mm and is placed near the commencement of the left common iliac artery (white arrow). **(B)**. A breach is visible in the arterial wall, and there is an overflow of contrast agents (white arrow).

**FIGURE 2:**
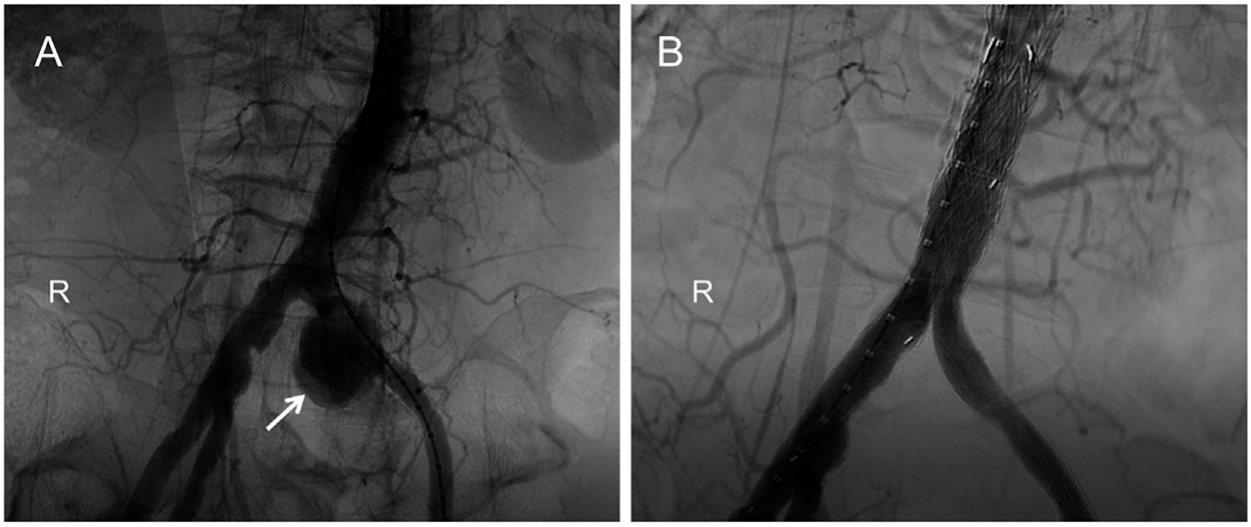
Digital subtraction angiography (DSA): **(A)**. The pseudoaneurysm was observed by contrast-medium overflow (white arrow). **(B)**. Postoperatively, angiography showed no contrast-agent spillage in the left common iliac artery.

A 67-year-old man, a local farmer specializing in sheep and swine production, was admitted to the Department of Vascular Surgery of the First Bethune Hospital of Jilin University in July 2019 after experiencing back pain for 3 days. For 1 month, the patient developed a fever with a maximum temperature of 39°C, which was alleviated with non-steroidal anti-inflammatory medicines (NSAIDs). The course of the disease was accompanied by fatigue and hyperhidrosis, but there was no joint discomfort. Medical and family history were unremarkable. The skin and sclera of the patient were not yellowish, nor were his abdominal muscles tense, tender, or creating rebound pain. The patient’s liver and spleen were not palpable beneath the ribs, nor was the entire abdomen. Percussion and auscultation in the abdomen were normal. Physical examination revealed a temperature of 36.3°C, heart rate of 78/min, respiratory rate of 18/min, and blood pressure of 146/80 mmHg. Laboratory examination showed the following results: C-reactive protein, 8.19 mg/L; erythrocyte sedimentation rate, 29 mm/1 h; white blood cell count, 5.95 × 10^9^/L; and hemoglobin, 127 g/L. All other laboratory tests were within normal limits. The *Brucella* tube agglutination test showed a titer of 1:400, and a solid and accurate diagnosis was made by isolating *Brucella* in the blood culture. We found pseudoaneurysm formation using computed tomography angiography (CTA) (Figures 1A,B). Accordingly, the patient was diagnosed with a brucellosis-related left common iliac artery pseudoaneurysm. The patient had no pseudoaneurysms elsewhere. To our knowledge, no previous report of a left common iliac artery pseudoaneurysm owing to brucellosis has been reported. Nonsurgical treatment of a common iliac artery pseudoaneurysm caused by brucellosis is ineffective, and it should be operated on as soon as possible. However, the patient underwent surgery while being infected systemically and then faced the possibility of reoperation and death following implant infection. Hence, a long-term and multi-course antibiotic treatment with combined antibacterial agents was required. Our patient was first treated for 6 weeks with doxycycline and rifampicin while preparing for emergency surgery at any time. After 6 weeks, the *Brucella* tube agglutination test was positive, and the blood culture of *Brucella* was negative. It represents a previous infection with *Brucella*. Endovascular aneurysm repair (EVAR) was performed under general anesthesia after the patient’s infection was controlled to prevent mortality and further complications from the left common iliac artery pseudoaneurysm. A TAG-covered stent graft (W. L. Gore & Associates, Inc., Flagstaff, AE, United States) was used to isolate the pseudoaneurysm. A 6F sheath (Terumo Corporation, Japan) was retrogradely inserted into the left common femoral artery intraoperatively. On digital subtraction angiography with a pigtail catheter, a pseudoaneurysm was identified (Figure 2A). Then, with the help of a Lunderquist^®^ Extra-Stiff Wire Guide (Cook Medical Inc., Denmark), a 22F sheath (W. L. Gore & Associates, Inc., Flagstaff, AE, United States) was introduced and a 26 mm × 12 mm × 18 cm TAG-coated stent-graft and a 16 mm × 12 mm × 12 cm endoprosthesis contralateral leg (W. L. Gore & Associates, Inc., Flagstaff, AE, United States) were implanted to isolate the pseudoaneurysm. In the final angiography, no endoleaks were identified. The contrast agent went smoothly through the left common iliac artery without leakage (Figure 2B). The anti-brucellosis medication was maintained following surgery due to possible recurrence, and the patient’s painful symptoms progressively subsided after 3 days. After surgery, the patient was discharged from the hospital. There were no reports of relapse in the 1-month, 6-months, 1-year, and 2-years follow-up assessments. The patient had no other endovascular complications.

## Discussion

Brucellosis is a zoonotic disease that is frequently transmitted from animals to humans, but is only rarely passed between humans (Colomba et al., 2012). Vascular pathological symptoms of brucellosis are infrequent (Willems et al., 2021). After bacterial colonization of the arterial wall (Amirghofran et al., 2011), the artery’s structure is damaged, which may result in life-threatening bleeding. Once ruptured, it can also be wrapped by surrounding soft tissues, resulting in the formation of pseudoaneurysms. Pseudoaneurysms produced by brucellosis are uncommon in clinical practice. Diagnosing and treating infected common iliac artery pseudoaneurysms, particularly those produced by *Brucella*, is more challenging than general pseudoaneurysms (Chiyoya et al., 2019). The incidence of positive blood cultures (particularly following antibiotic therapy) is clinically insignificant, and markers such as erythrocyte sedimentation rate, C-reactive protein (al-Kassab et al., 1991), and procalcitonin are not specific for infected pseudoaneurysms (Kayaaslan et al., 2016; Liu and Zhao, 2017). The signs and symptoms of the patient were not specific (Harman et al., 2004). Patients in some cases were asymptomatic. Owing to the fact that *Brucella* reproduces primarily in the cells of the human reticuloendothelial system, it is difficult to treat and relapses often. If pseudoaneurysms are not treated promptly, they might rupture at any time. As a result, it must be operated upon as soon as possible. Traditionally, aneurysmectomy, local debridement, and graft replacement have been used to treat this condition (Chakfé et al., 2020). Under general anesthesia, the patient had a major incision to reveal and excise the pseudoaneurysm and replace it with an artificial blood vessel. Pseudoaneurysms lack a full wall, making surgery challenging, posing a high risk of rupture, resulting in significant surgical stress and a protracted postoperative recovery time. While endovascular repair involves minimal trauma, has a low complication rate, and allows for rapid postoperative recovery (Willems et al., 2021), it has stringent criteria for correct stent placement and sealing. Primary treatment, in our opinion, should always be the first option in endovascular surgery. Because endovascular repair does not require anatomical or arterial incision, it significantly streamlines the surgery process. This method results in less bleeding and trauma; is well tolerated by the patient and is safe and effective; results in fewer problems; has a quicker recovery; and has other benefits that traditional surgery cannot match (Goodney et al., 2011). The primary advantage of endovascular repair for an infected left common iliac artery pseudoaneurysm is that it minimizes the risk of infection spreading. If endovascular repair is unsuccessful, conventional surgery to remove the iliac artery and rebuild arterial access can be undertaken. However, endograft infection and difficulty with secondary intervention are common issues for both intracavitary and open conventional surgery. Infected endografts are very difficult to treat, and often have poor prognosis (Laohapensang et al., 2017). Therefore, surgical therapy should begin as soon as the existing infection is managed. As a result, antibiotics must be used in combination. The World Health Organization recommends a 6-weeks course of doxycycline (100 mg, bid) and rifampicin (600–900 mg/d) (Fillmore and Valentine, 2003). Reasonable and standardized application of antibiotics can play a key role in the effective control of *Brucella*, thereby reducing the possibility of pseudoaneurysms in other blood vessels. Once the pseudoaneurysm appears, it should be treated as soon as possible to avoid aggravation. Therefore, regular physical examinations are necessary. To assess if a patient can wait for surgery following infection management, we consider two factors: 1) repeat CT to detect whether the pseudoaneurysm body has increased further; and 2) determine whether the patient’s symptoms such as back pain and fever have deteriorated. Following a repeat CT examination, our patient’s pseudoaneurysm body did not expand, and the patient’s symptoms progressively improved with anti-infective medication. In preparation for emergency surgery, we make every effort to complete an adequate antibiotic course. No bacterial growth was seen in blood cultures. After the patient’s infection was managed, EVAR was conducted. Antibiotic therapy was continued for 6 weeks following surgery. We recommend CT examination every 6 months. Although endovascular therapy has established itself as a safe and successful alternative to open surgery in the treatment of pseudoaneurysms, there is still a dearth of experience in this area. Simultaneously, medication therapy for brucellosis is crucial. Thus, we presented this case report to increase awareness of the condition, decrease the rate of missed diagnoses and misdiagnoses, and promote active and successful treatment and improve prognosis. Therefore, in individuals with a history of contact with cattle and sheep, long-term unexplained fever, and back and abdominal pain, *Brucella-*infection induced common iliac artery pseudoaneurysm should be considered in the differential diagnosis, and timely blood culture, antibody testing, and CTA, as well as other related checks should be performed to avoid life-threatening delays in treatment.

## Data Availability

The original contributions presented in the study are included in the article/Supplementary Material, further inquiries can be directed to the corresponding authors.
